# Hybrid blade and locking plate fixation for proximal humerus fractures: a comparative biomechanical analysis

**DOI:** 10.1186/s12938-018-0447-y

**Published:** 2018-01-25

**Authors:** Ali Jabran, Chris Peach, Zhenmin Zou, Lei Ren

**Affiliations:** 1 School of Mechanical, Aerospace and Civil Engineering, University of Manchester, Sackville Street, Manchester, M13 9PL UK; 2 Department of Shoulder and Elbow Surgery, University Hospital of South Manchester, Southmoor Road, Wythenshawe, Manchester, M23 9LT UK

**Keywords:** Biomechanical analysis, Proximal humerus fractures, Plate fixation, Blade plate, Locking plate

## Abstract

**Background:**

Open reduction and internal fixation of proximal humerus fractures can be difficult to achieve adequate, complication free results due to osteopenia of the proximal humerus and unstable fracture patterns. This study aimed to compare the biomechanical properties of a novel hybrid fixed angle blade plate (Fx plate) with an established fixed angle locking plate (PHILOS plate).

**Methods:**

A two-part fracture was simulated in synthetic composite humeri by creating a transverse osteotomy and 10 mm fracture gap at the surgical neck. After treating the fractures with either an Fx plate or a PHILOS plate, humeral head was fixed and the shaft was displaced in a cantilever fashion. For elastic tests, loading was along the frontal and sagittal plane to achieve varus/valgus and extension/flexion, respectively. In plastic tests, loading was in a varus direction to determine the constructs’ resistance to varus collapse.

**Results:**

In elastic tests, both construct types had higher peak load and stiffness in extension/flexion than varus/valgus. Fx plate constructs were significantly stiffer than PHILOS constructs in varus/valgus (mean: 7.590/6.900 vs. 6.609/6.091 N/mm; p < 0.001 for both) but significantly less stiff in extension/flexion (8.770/9.541 vs. 9.533/9.997 N/mm; p < 0.001 for extension, p < 0.05 for flexion). In varus plastic tests, significantly higher peak loads were reported for Fx plate than PHILOS (134.391 vs. 115.531 N; p < 0.001).

**Conclusions:**

In this fracture gap model, humeri implanted with a novel Fx plate provided higher varus/valgus stiffness but lower extension/flexion stiffness than a more traditional proximal humeral locking plate design (PHILOS).

## Background

Fractures of proximal humerus are the third most common fractures of the human body, representing 5% of all fractures in all age groups [1, 2]. They are also the third most common fractures in patients over the age of 65 years and are mostly linked to osteoporosis [3]. The majority (85%) of proximal humerus fractures are minimally displaced and can be treated with good functional outcomes non-operatively [4–9]. Management of severely displaced fractures such as three- and four-part fractures remains a surgical challenge due to poor bone quality, forces from the rotator cuff and the increased risk of avascular necrosis caused by humeral head devascularisation. Managing these fractures conservatively lead to poor outcomes, mainly secondary to bony deformity and stiffness [9, 10]. Due to conflicting evidence, controversy still lies around whether an operative or a non-operative approach leads to the best outcomes for patients. One possible reason for the observed increase in the incidence of open reduction and internal fixation procedures could be due to design innovation of fracture implants. Several design philosophies and technologies have emerged including locking and, more recently, hybrid blade plate designs.

Locking plates rely on screws being able to lock into the threaded plate, resulting in a fixed-angle fixation. This construct aims to improve fracture stability and is reliant on the bone-screw interface instead of the bone-plate interface. In the clinical setting, however, their performance varies as several implant-specific problems have arisen. Clinical studies report a high rate of complications such as varus deformity, screw penetration into the joint, cut-out, need for revision surgery, malreduction, avascular necrosis and tuberosity displacement [1, 11–16].

Blade plates allow insertion of the blade often at the humeral head’s medial calcar region. Blades are inserted to further buttress the bone-plate construct by supporting the humeral head from varus collapse. The fundamental reason behind using a blade instead of the screw is that the former provides the construct with a larger surface area than the latter. Theoretically, this is beneficial not only for support in fracture fixation but also for avoiding cut-out, a common complication associated with screw-based fixations. In general, blade plates have become less popular as they were unable to counter the large coronal plane bending moment. When a blade plate is used, it appears to be associated with poor clinical outcomes [17]. Results from the clinical and biomechanical studies of blade plates are also found to vary considerably, making it difficult to derive a generalised conclusion [18–21].

Merging the two types of plates, a concept of hybrid plate has recently emerged, offering implantation of both blades and locking screws to a single plate with the aim of reaping the benefits of both and compensate for each other’s disadvantages. Thus, the purpose of this study was to compare, in a synthetic bone model, the biomechanical stability of two-part fractures of the surgical neck treated with the novel hybrid fixed angle blade plate with those treated with a fixed angle locking plate.

Our hypothesis was that the new hybrid blade plate and locking fixation construct would exhibit superior biomechanical performance compared to the standard locking plate fixation.

## Methods

### Specimen preparation

Ten left synthetic humeri (model 1028; Pacific Research Laboratories, Vashon, WA, USA) were obtained. To allow secure clamping of the humeri during the tests, they were potted in cement blocks. To achieve this, each humeral head was placed inside a 10 cm cubic mould such that the section from the head apex and 4 cm distal was inside the mould. Sides of the mould were parallel to the sagittal and the frontal plane. Once the humerus was in the correct place, a mixture containing general purpose (Portland limestone) cement, rapid mix cement and water at a ratio of 4:1:2.5 by volume, was prepared and poured into the mould. Care was taken to ensure that the cement did not cover the region where the implant would be inserted. At least 48 h were required for a block to dry sufficiently and be ready for removal from the mould. Upon removal, a transverse cut was made 21 cm from humeral head apex to discard the distal end of the humerus.

Specimens were split into two groups of five; one group was implanted with a 90 mm PHILOS plate (Synthes, Paoli, Pennsylvania, USA) and the other with an 80 mm Equinoxe Fx plate (Exactech, Gainsville, FL). As per manufacturers’ guidelines, plates were implanted approximately 30 and 12 mm distal to the superior greater tuberosity, respectively. All implantations were performed using the surgical instruments provided by manufacturers by a senior orthopaedic consultant surgeon.

**Fig. 1:**
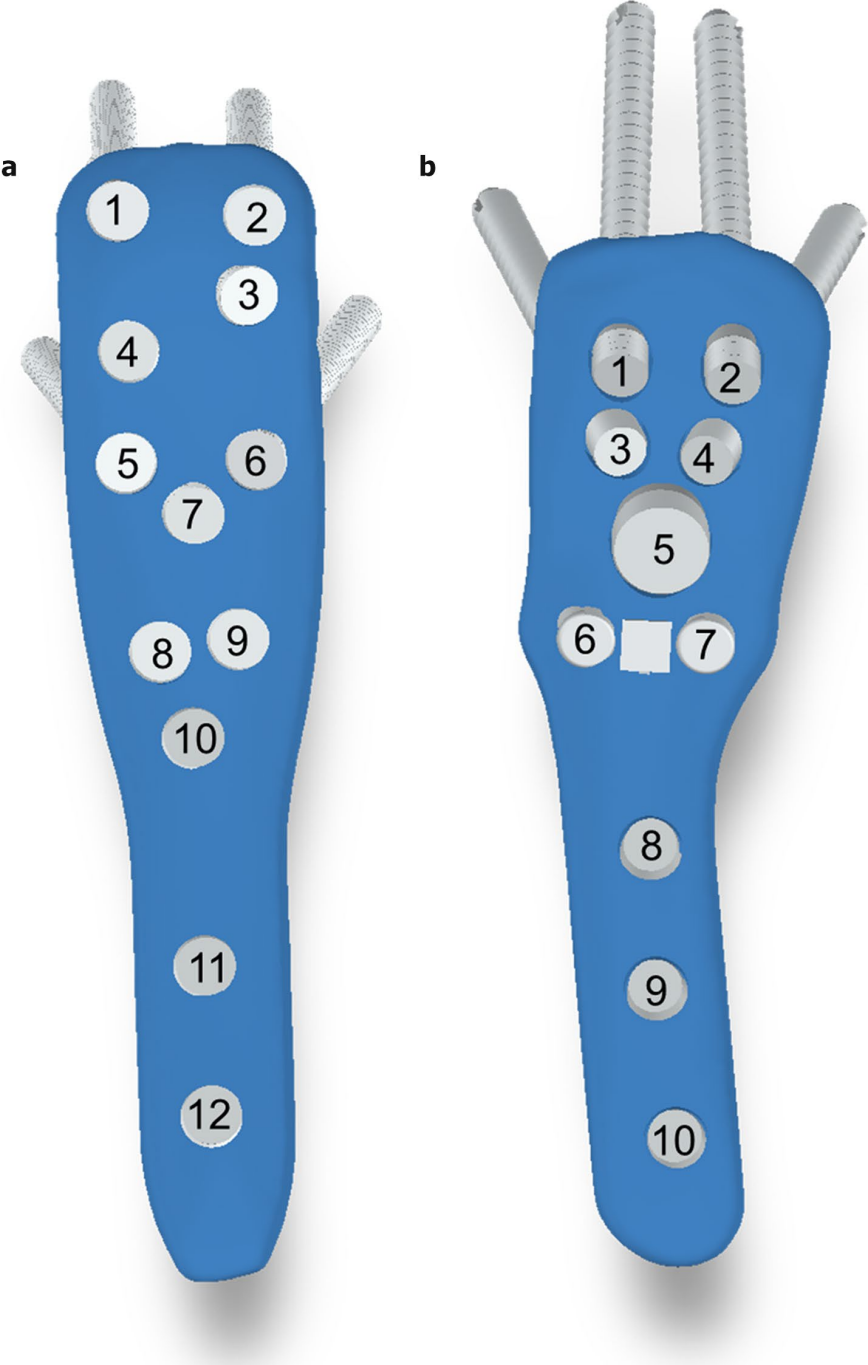
Numbering of screws and blade on DePuy Synthes PHILOS plate (**a**) and the Fx plate (**b**)

For PHILOS, all but the screw above the two calcar screws (Fig. 1a, screw hole 7) were filled using 3.8 mm locking screws. A pair of 40 mm screws were inserted in the most proximal screw holes (1 and 2) followed by a 42 and a 50 mm screw at screw hole 3 and 4. Screw holes 5 and 6 were filled with screws of length 40 mm while screw holes 8 and 9 were with 50 mm ones. Moving distally, the three shaft screws were 32, 30 and 30 mm long.

Likewise, for Fx, all but the calcar screws themselves (Fig. 1b, screw holes 6 and 7) were filled. There, a 45-mm blade was inserted instead. A 6.5 mm diameter locking screw of length 50 mm was inserted into screw hole 5 while other screws were 3.8 mm. Screw holes 1–2 and 3–4 were treated with pairs of 44 and 23 mm locking screws. 26 mm cortical locking screws were inserted into screw holes 8 and 10 while a 32 mm non-locking compression screw was inserted in screw hole 9.

These lengths were determined in pilot experiments by using the depth gauge until resistance from subchondral bone was felt, allowing the maximum purchase. Upon implantation, a two-part, unstable fracture pattern was simulated, by creating a transverse osteotomy at the surgical neck (50 mm from the humeral head apex) with a 10-mm fracture gap.

### Biomechanical testing

**Fig. 2:**
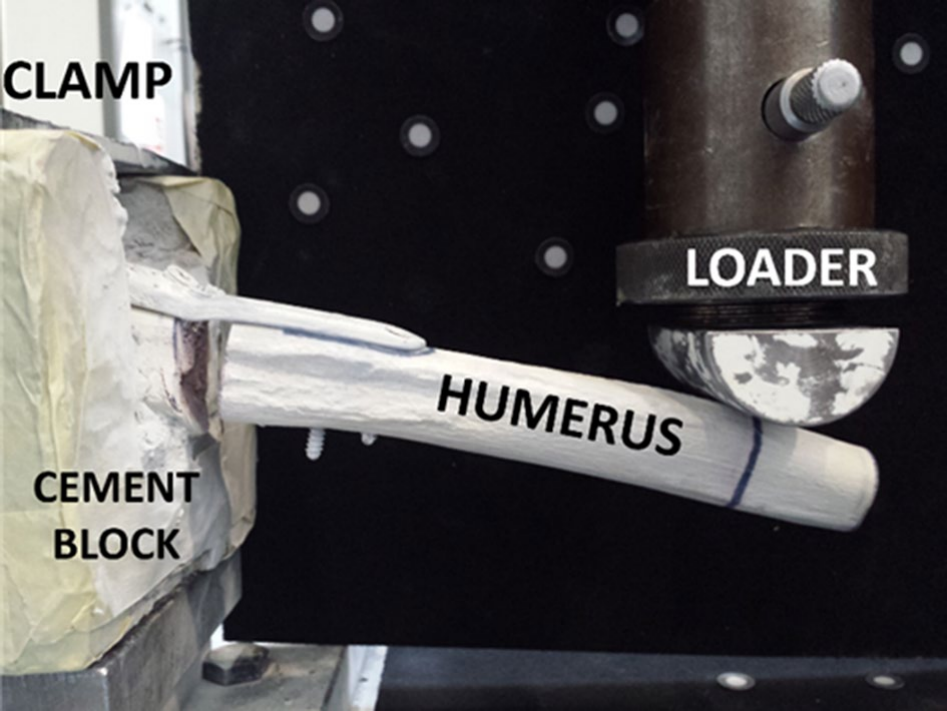
Experimental setup for performing bending tests, shown with Fx plate in varus

For testing the specimens were placed in a uniaxial Instron 4500 material testing machine (Instron, Canton, MA, USA), with the cement block clamped to machine’s base and shaft perpendicular to a semi-cylindrical loader (Fig. 2).

First, elastic testing was performed, for which each specimen was subjected to five trials of varus, valgus, extension and flexion bending. This was achieved by turning the cement block to the corresponding planes. For each of the four directions, loads were applied 12 cm distal to the fracture site in a cantilever fashion under displacement control at a rate of 1 mm/s up to 5 mm of actuator displacement. Based on pilot experiments, this 5-mm displacement was found to be well within the linear elastic region of both plate constructs for 1 mm/s displacement rate. From the load–displacement data recorded, load at 5 mm and the stiffness (K, gradient of the linear portion of the force–displacement plot) were calculated for each specimen. From these, the mean value of stiffness (K) and load at 5 mm (F_5_) for five specimens were calculated in each construct group.

In order to investigate constructs’ resistance to varus collapse, it was important to subject them to varus displacements well within the constructs’ plastic region. Therefore, after completion of the elastic testing in the four load directions, specimens were set in the varus position to begin plastic tests where they were loaded at 0.05 mm/s from 0 to 15 mm displacement and the crosshead was arrested for 8 min. Then, at the same displacement rate, specimens were loaded from 15 to 30 mm displacement, well within their plastic region. Load at 15 mm before (F_15a_) and after (F_15b_) the eight-minute intermission and load at 30 mm (F30) were determined from the load–displacement data. Pilot experiments revealed that at 0.05 mm/s displacement rate, 30 mm displacement was sufficient to cause plastic deformation of both plate constructs.

### Statistical analysis

SPSS 22.0 statistical analysis software (IBM, NY, USA) was used to perform statistical analysis of the obtained data. Statistical significance of the effect of different plates on constructs’ stiffness and load values was determined via a linear mixed model approach by taking intra- and inter-subject variability into account. In the analysis, the fixed effect was the plate type while the specimens and trials were the random effects. The dependent variables were K and F_5_ for elastic tests and F_15a_, F_15b_ and F_30_ for plastic tests. Differences between each pair were tested using Fisher’s least significant difference (LSD) multiple comparison based on the least-squared means. The statistical significance was set at p < 0.05.

## Results

**Fig. 3:**
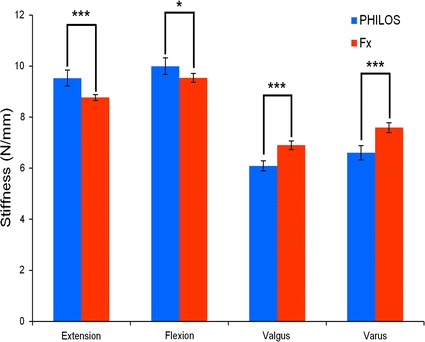
Mean stiffness (S) for PHILOS and Fx plate constructs during elastic loading of 5 mm cantilever displacement in extension, flexion, valgus and varus directions. For each direction, stiffness is presented as the mean for five specimens (25 trials) in each construct group. A single asterisk (*) indicates p ≤ 0.05; and triple asterisks (***) indicate p ≤ 0.001

**Fig. 4:**
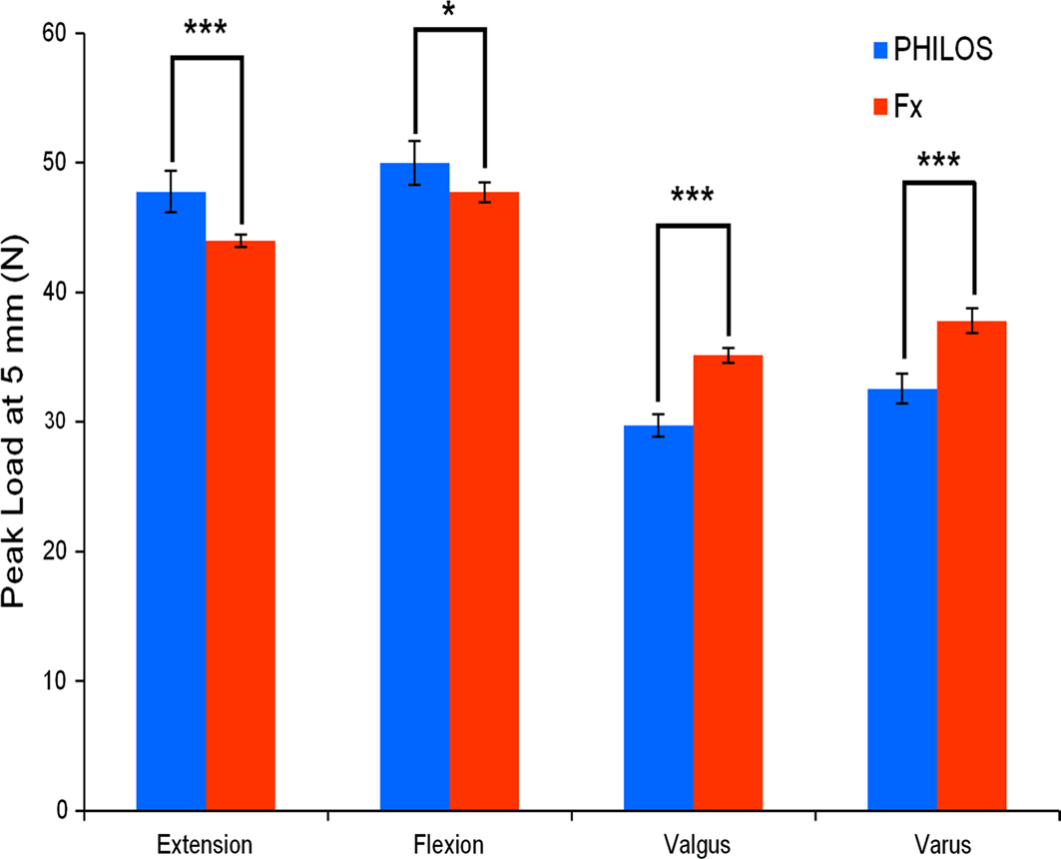
Mean peak load (F_5_) for PHILOS and Fx plate constructs during elastic loading of 5 mm cantilever displacement in extension, flexion, valgus and varus directions. For each direction, peak load is presented as the mean for five specimens (25 trials) in each construct group. A single asterisk (*) indicates p ≤ 0.05; and triple asterisks (***) indicate p ≤ 0.001

No implant failure or cut-out was noted for any of the construct groups in either elastic or plastic test. In the elastic tests, for both fixation constructs, the directions could be ranked in the following order of decreasing construct stiffness: flexion, extension, varus and valgus (Fig. 3). The PHILOS plate constructs demonstrated significantly higher stiffness than Fx constructs in extension (p < 0.001) and flexion (p = 0.025) with mean stiffness values 8.69 and 4.77% higher (Table 1). This is the conversely, the Fx plate constructs demonstrated significantly higher stiffness than PHILOS constructs in varus and valgus (p < 0.001 for both), with mean stiffness values 14.85 and 13.27% higher, respectively. Expectedly, these trends were consistent across F_5_ values obtained (Fig. 4).

**Table 1:** Mean stiffness (K) and load values (F) for all specimens of both plate constructs in each loading direction with their respective standard deviations (S.D.) and p values from pairwise statistical comparisons

Loading direction/variable	Fx plate construct ± S.D.	PHILOS plate construct ± S.D.	p value
Extension			
K (N/mm)	8.770 ± 0.156	9.533 ± 0.286	< 0.001
F_5_ (N)	43.979 ± 0.596	47.749 ± 1.510	< 0.001
Flexion			
K (N/mm)	9.541 ± 0.221	9.997 ± 0.298	< 0.05
F_5_ (N)	47.711 ± 0.775	49.981 ± 1.569	< 0.05
Valgus			
K (N/mm)	6.900 ± 0.200	6.091 ± 0.181	< 0.001
F_5_ (N)	35.131 ± 0.617	29.746 ± 0.815	< 0.001
Varus			
K (N/mm)	7.590 ± 0.196	6.609 ± 0.256	< 0.001
F_5_ (N)	37.792 ± 0.990	32.561 ± 1.075	< 0.001
F_15a_ (N)	84.470 ± 1.547	75.590 ± 3.049	< 0.001
F_15b_ (N)	79.304 ± 2.507	71.558 ± 3.303	< 0.01
F_30_ (N)	134.391 ± 3.574	115.531 ± 6.336	< 0.001

K and F_5_ are the stiffness and peak load values during elastic tests, respectively. F_15a_ and F_15b_ are loads at 15 mm before and after eight-minute intermission and F_30_ is the load at 30 mm

**Fig. 5:**
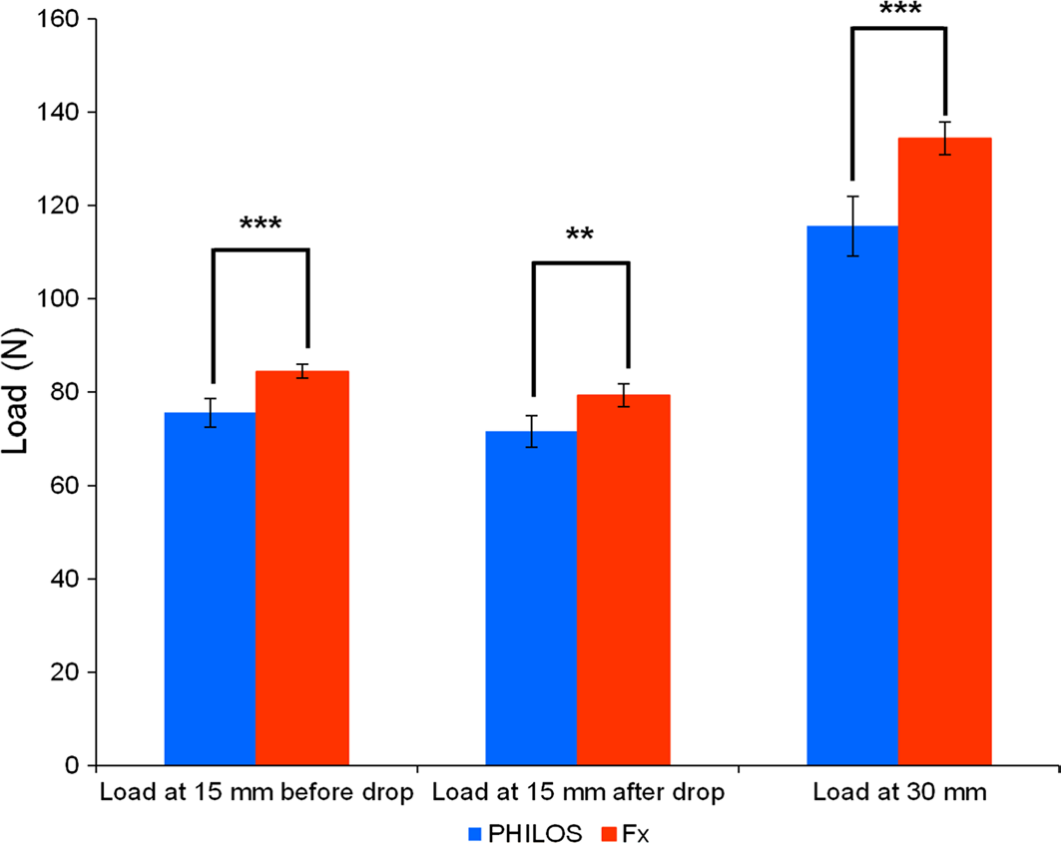
Mean load for PHILOS and Fx plate constructs during plastic loading at 15 mm displacement before (F_15a_) and after (F_15b_) eight-minute intermission and at 30 mm displacement (F_30_). Load is presented as the mean for five specimens (5 trials) in each construct group. A single asterisk (*) indicates p ≤ 0.05; and double asterisks (**) indicate p ≤ 0.01

**Fig. 6:**
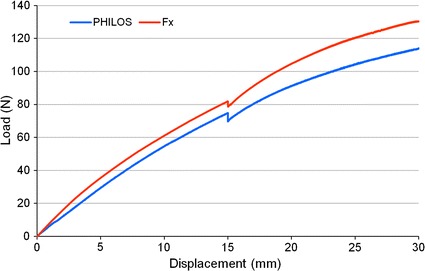
Typical load–displacement curves at load point for PHILOS and Fx plate constructs during plastic loading. A drop of 4–5 N in load is noted at 15 mm displacement due to the stress relaxation of construct during the eight-minute intermission

In plastic varus failure tests, Fx plate constructs’ load at 30 mm were significantly higher (p < 0.001) than those of PHILOS, with former’s mean values being 134.39 N compared to latter’s only 115.53 N, an increase by 16.32% (Fig. 5). Both construct groups were found to retain their structural integrity as there was no observation of screw pull-out. Temporal stress decay was observed during the eight-minute intermission, owing to stress relaxation, a phenomenon commonly exhibited by viscoelastic materials such as polyurethane (a primary constituent of synthetic humeri) when under constant strain (Fig. 6). As a result of stress relaxation, an approximately 4–5 N drop in load (the difference between F_15b_ and F_15a_) was recorded on the load–displacement plot at abscissa of 15 mm.

## Discussion

The PHILOS plate is one of the most widely studied proximal humerus fixation systems in biomechanical literature, introduced typically as the representative of the broad category of locking plates. On the contrary, to our knowledge, no such study exists for the Fx plate constructs yet or to compare the performance of this new concept of hybrid blade plates. In addition to being the first in vitro biomechanical study on hybrid blade plates, the current study has two novel contributions to the biomechanical literature.

One significant finding of the current study was that the Fx offered stiffer constructs in varus and valgus bending than the PHILOS plate. The implications of this finding are of key importance given that the varus malreduction and displacement has been reported to be one of the most common complications associated with locking plates [22–24]. By exhibiting higher stiffness in varus/valgus, Fx plate appears to have beneficial characteristics that could reduce this severe complication for patients. One possible reason is the position of the blade. The blade begins near the fracture gap and crosses the calcar region, a region critical for humeral head’s stability against varus collapse [25, 26]. Its increased surface area might significantly improve the support in this important region. This finding is in contrast to the study by Siffri et al. which reported statistically similar varus bending stiffness between a locking plate and a traditional non-locking blade plate. Also, Gillespie et al. subjected cadaveric humeri to 20° of abduction from vertical [27] and the mean stiffness value for non-locking blade plate was found to be 12% higher than locking plate but with no statistically significant difference.

The second significant finding of this study was that the PHILOS plate was stiffer than the Fx plate in extension and flexion bending, thereby only partially confirming our hypothesis. Everyday movements of the glenohumeral joint and specifically the humerus are complex, involving a combination of varus, valgus, extension and flexion bending, as well as torsion and compression. When comparing the performance of bone plate construct, extension and flexion bending stiffness should also be accounted, despite the high prevalence of varus failure. In this regard, the stiffer constructs achieved by PHILOS plate under extension and flexion bending is noteworthy, since in everyday movements such as arm extension and flexion, the stability in these two directions also contribute to the potential failure of the bone-plate construct. The difference in the performance of the two plates may also be due to their screw hole designs. A non-locking screw had to be used in Fx plate’s hole 9 since the plate does not allow insertion of a locking screw there. In PHILOS, all screws were locking and this may have potentially affected the relative stiffness of the two plate constructs.

Based on previous biomechanical comparisons between locking plates and non-locking blade plates, the locking plate may also be stiffer than the Fx plate during torsion tests. Weinstein et al. [21], for example, compared the torsional performance of a locking plate with an angled blade plate for treatment of three-part fractures on cadaveric humeri. Mean initial torsional stiffness was reported to be significantly higher for the locking plate than a blade plate. Siffri et al. [28] seem to provide ample support to this conclusion. Like Weinstein et al. they also applied torsional loading and reported significantly less fixation loosening for locking plate than the blade plate, at least for cadaveric humeri.

In elastic tests, the 5-mm displacement limit was set purely to keep the specimens well within their elastic region, allowing testing in all four directions and subsequent plastic testing. Results from plastic testing show that the biomechanical superiority of the Fx plate constructs over PHILOS plate constructs under varus loading, in terms of construct peak loads and stiffness, holds true beyond the elastic region. Despite the drop in the load in the course of the eight-minute intermission, Fx specimens remained to be stronger than the PHILOS.

Cantilever loading has been used previously in the literature, often to achieve a bending moment of 0–7.5 Nm at the fracture site [28–33]. Comparable bending loads were applied by Chow et al. and Weeks et al. who performed cantilever bending on the basis of a biomechanical study by Poppen and Walker, with the aim of replicating the supraspinatus forces on bone-plate constructs during the early stages of healing under shoulder immobilisation support [29, 32, 34]. Mechanically, this loading is comparable to humeral immobilisation followed by a varus force acting directly at the supraspinatus insertion site. A similar range of bending moments (at fracture site) was achieved in our study during elastic loading (0–4.8 Nm). For the plastic loading, however, these bending moments reached up to approximately 17 Nm for Fx plate constructs and 14 Nm for PHILOS plate constructs. Despite these loadings, no implant failure was reported.

The choice for the use of synthetic humeri was based on a recent biomechanical study where the same polyurethane foam humeri as those used in this study were tested along with human cadaveric humeri [35]. In the study, the results for cadaveric specimen had large variations, due to their inherent biologic variability. Huff et al. were able to draw similar conclusions from the testing of cadaveric and synthetic specimens and recommended synthetic humeri for future studies.

A disadvantage of our experimental procedure, and in fact of most biomechanical studies in literature, is that the in vivo forces acting on the humerus are unlikely to be unidirectional. Human shoulder movement is extremely complex. Designing and conducting in vitro biomechanical tests that accurately simulate in vivo conditions of a joint with such a high degree of complexity as the glenohumeral joint is in itself a major challenge in the advancement of investigations into the treatment of proximal humerus fractures.

We approached this problem by loading the specimens along the two main anatomical planes and in the four directions sequentially, instead of simultaneously, to make the results simpler to interpret and yet clinically relevant. Nevertheless, caution should be exercised when extrapolating these experimental laboratory findings to the clinical situation.

One possible way to tackle this limitation is to load the humerus in the magnitude and direction of the resultant force of glenohumeral joint for a given shoulder movement which takes into consideration the muscles forces, bone-to-bone forces and connective tissue forces. Even then, however, there remains inaccuracy in loading conditions because the glenohumeral joint forces on their own do not fully depict the in vivo scenario. The humerus has insertion points for most shoulder muscles including the deltoid, infraspinatus, supraspinatus, subscapularis and pectoralis major muscles, all of which pull at a different region of the humerus at different stages of shoulder movements. More accurate simulation of in vivo conditions would be possible by first obtaining a cadaveric shoulder complex with muscles attached and then pulling individual muscles to create desired movements. This type of testing has already been conducted in the literature, notably by Voigt et al. and her colleagues [36–38]. They used a robot-assisted shoulder simulator which allowed differentiated application of defined muscle forces (such as that of the rotator cuff muscles) along with their physiological lines of action and in proportion to their respective physiological cross-sectional areas. Studies such as these involve cadaveric humeri, thus the issue of inter-specimen variability have to be taken into consideration. In Voigt’s et al. study, input values were from previous studies, however, measurement of in vivo biomechanical contribution of individual muscle during shoulder movement is challenging and is a hot topic in the literature [39–41].

## Conclusions

This study was the first in vitro biomechanical comparison of hybrid blade plate and locking plate. It was found that while the hybrid plate demonstrates superior biomechanical characteristics in varus and valgus, it is inferior to the locking plate extension and flexion. Since the commonest mode of failure is varus collapse and subsequent screw cut out, this data suggests that the hybrid blade plate is better able to prevent them. However, everyday movements are complex and bone-plate construct’s stability in all four directions can contribute to fixation failure. Thus, further clinical studies are required to investigate the implications of hybrid blade plate’s inferior extension and flexion bending stiffness under more complex movements.
